# Automatic volumetry on MR brain images can support diagnostic decision making

**DOI:** 10.1186/1471-2342-8-9

**Published:** 2008-05-23

**Authors:** Rolf A Heckemann, Alexander Hammers, Daniel Rueckert, Richard I Aviv, Christopher J Harvey, Joseph V Hajnal

**Affiliations:** 1 Division of Neurosciences and Mental Health, Imperial College London, Hammersmith Campus, Du Cane Road, London, UK; 2 Imaging Sciences Department, Clinical Sciences Centre, Imperial College London, Hammersmith Campus, London, UK; 3 Department of Computing, Imperial College London, South Kensington Campus, London, UK; 4 Department of Diagnostic Imaging, Sunnybrook Health Sciences Centre, Toronto, Canada; 5 Department of Imaging, Hammersmith Hospital, Du Cane Road, London, UK

## Abstract

**Background:**

Diagnostic decisions in clinical imaging currently rely almost exclusively on visual image interpretation. This can lead to uncertainty, for example in dementia disease, where some of the changes resemble those of normal ageing. We hypothesized that extracting volumetric data from patients' MR brain images, relating them to reference data and presenting the results as a colour overlay on the grey scale data would aid diagnostic readers in classifying dementia disease versus normal ageing.

**Methods:**

A proof-of-concept forced-choice reader study was designed using MR brain images from 36 subjects. Images were segmented into 43 regions using an automatic atlas registration-based label propagation procedure. Seven subjects had clinically probable AD, the remaining 29 of a similar age range were used as controls. Seven of the control subject data sets were selected at random to be presented along with the seven AD datasets to two readers, who were blinded to all clinical and demographic information except age and gender. Readers were asked to review the grey scale MR images and to record their choice of diagnosis (AD or non-AD) along with their confidence in this decision. Afterwards, readers were given the option to switch on a false-colour overlay representing the relative size of the segmented structures. Colorization was based on the size rank of the test subject when compared with a reference group consisting of the 22 control subjects who were not used as review subjects. The readers were then asked to record whether and how the additional information had an impact on their diagnostic confidence.

**Results:**

The size rank colour overlays were *useful *in 18 of 28 diagnoses, as determined by their impact on readers' diagnostic confidence. A *not useful *result was found in 6 of 28 cases. The impact of the additional information on diagnostic confidence was significant (*p *< 0.02).

**Conclusion:**

Volumetric anatomical information extracted from brain images using automatic segmentation and presented as colour overlays can support diagnostic decision making.

## Background

During the last four decades, numerous efforts have been directed at developing computer-assisted diagnosis and making such methods usable in practice [1]. The promise is that computer-assisted diagnosis will increase screening capacities, reduce the problem of observer dependence and increase diagnostic accuracy in imaging. Most of these developments are centred around algorithms that identify patterns suggestive of disease in two-dimensional (2D) images. Some success has been achieved in mammography (detecting massses and clustered microcalcifications), chest radiography (lung nodules and vertebral fractures), and magnetic resonance angiography (intracranial aneurysms) [2]. Implementing a technical system that performs generic pattern recognition is fraught with algorithmic challenges. The conjecture behind our work is that decision support systems which complement human pattern recognition capabilities, rather than attempting to supplant them, will prove to be more useful and could have a major impact on clinical radiology and imaging research.

Brain imaging lends itself particularly well to investigating and developing this potential. A large body of published research exists on the computational problems of tissue classification, registration and atlas label propagation. The resulting capabilities have been applied in neurological science, for example for discovering relationships between brain structure and neurological function. The potential benefits of these advanced image processing methods for clinical diagnostic radiology are underexplored in comparison. In the current paradigm, clinical diagnosis generally relies on visual assessment of 2D sections calculated from the data, even when three-dimensional (3D) volumes have been acquired. The expert diagnostic reader forms a 3D mental representation of a patient's anatomy based on pixel intensities and compares this representation with a learned model of normal anatomy. The validity of the resulting interpretation depends on the reader's experience and the type and stage of pathology present. Neurodegenerative disease can be particularly difficult to detect, with low levels of interobserver agreement, especially when comparing inexperienced with experienced readers [3]. In some entities, notably Alzheimer disease (AD), typical findings can be difficult to distinguish from dynamic alterations in normal ageing [4].

When pathological change is complex, distributed, and symmetric, the relatively poor performance of human observers is in part explained by the difficulty of assessing structure volumes from sectional images. A volume increase by a factor of two in a hypothetical spherical structure would be reflected by an increase of the radius of only circa 26 %. An arbitrary change in the through-plane dimension of a structure goes completely unnoticed on a single sectional image.

Image data displays that combine information from different modalities by overlaying false colour are commonplace [5]. Structural grey scale ultrasound images, for example, are routinely supplemented in clinical practice with functional information determined using Doppler blood flow measurements and presented as colour overlays. Similarly, other combinations of structural and functional imaging modalities are used to produce "fusion" images. Reports where such an approach has been used to enhance the diagnostic value of structural images with computationally derived comparison measures [6] are surprisingly scarce.

A procedure that could reliably extract volumetric information from a target image and present it in an integrated fashion could complement human capabilities in diagnostic imaging. We hypothesized that using automatic segmentation and comparative volumetric information presented as colour overlays on sectional images can improve confidence in the diagnosis of AD. We tested this hypothesis in a forced-choice reader study.

## Methods

### Study data

MR images from a cohort of 36 subjects (age 72.5 ± 8 years), who had taken part in a previous study on AD undertaken by the Oxford Project to Investigate Memory and Aging (OPTIMA), were available for this work. Seven of these subjects (age range 57 to 82 years) were classed as affected by AD according to NINCDS criteria [7] (possible disease, n = 2; probable disease, n = 5). Beyond this classification, no individual data on the clinical status was available. The remaining 29 subjects were of comparable age (range 54 to 77 years) and did not meet clinical criteria for AD. In the OPTIMA study, volume images with a spatial resolution of 1 *mm*^3 ^had been acquired using T1-weighted sequences. Participants in the study had given informed consent to the scientific use of their clinical and imaging data. OPTIMA protocols have been described previously [8] and were approved by the Central Oxford Research Ethics Committee.

### Anatomical segmentation

The study data sets were segmented into anatomically defined regions of interest (ROI) through atlas label propagation. The atlas used was a labelled MR data set obtained from the Internet Brain Segmentation Repository, provided by the Center for Morphometric Analysis (CMA) at Massachusetts General Hospital [9]. The MR image had been segmented manually into 43 structures by a trained expert using a guided procedure [10]. The segmentation was supplied as a separate label set that corresponded spatially with the MR image, with voxel fill values linked to the name of a region via a lookup table.

The label propagation procedure was equivalent to the single-atlas method described in [11], consisting of skull stripping, affine registration and nonrigid registration using multi-resolution free-form deformations down to a control point spacing of 2.5 *mm*. The output was used to transform the atlas label set into the space of the target subject.

### Comparative volumetry

**Figure 1:**
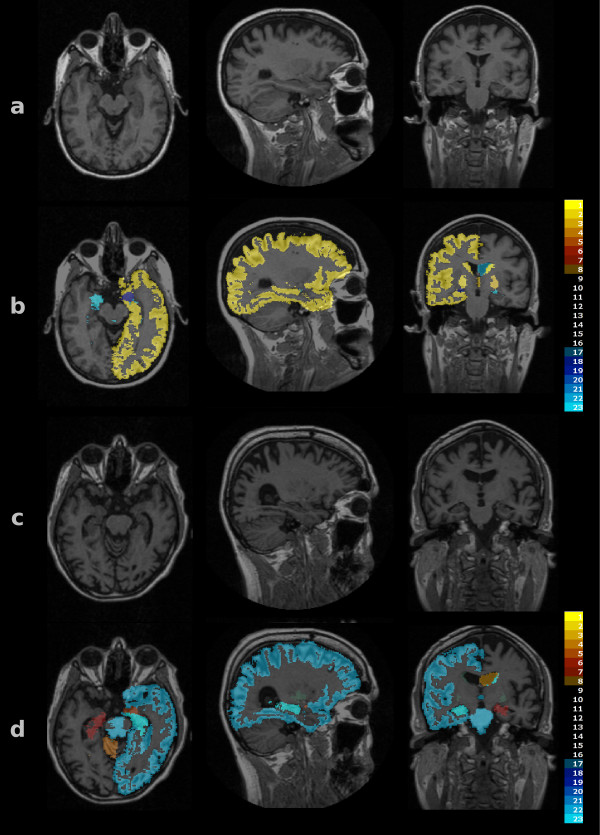
**Colour overlays**. Images of test subjects as shown to readers. Row a: grey scale images of a control subject. Row b: same sections as shown in a) with a colour overlay indicating the size rank of the labelled structures. Most structures are in the medium range (transparent overlay). The left cortex is among the largest, as shown by the yellow label. Row c: subject with AD. Row d: same sections as c) with colour overlay. The left cortical volume is small, while the right lateral ventricle is large.

The seven AD subjects, together with seven randomly chosen normal study subjects from the OPTIMA cohort, were used as the test subject cohort. The remaining 22 normal study subjects were used as the reference cohort, providing a measure of normality that the test subjects could be compared against. For each ROI, and for each test subject in turn, rank tables were generated, showing the relative size of the test subject's ROI as a rank number from 1 to 23 (22 control and one test subject). Colour overlays were generated for each test subject using pre-defined lookup tables. The colour scheme used was as follows: ranks 1 to 4 were shown in shades of yellow, 5 to 9 in shades of red, 10 to 13 fully transparent, 14 to 18 shown in shades of blue and 19 to 23 in shades of cyan (Figure 1). The highest and lowest ranks were thus coloured brightly, while structures in the mid-range of the size rank order were not coloured at all.

### Visual review

All 14 test subjects were reviewed by a neuroradiologist and a general radiologist. They were informed of the subjects' age, sex and number of AD (7/14) and control cases (7/14) in the group, but were blinded to the individual diagnosis and other clinical information. Readers made a forced-choice classification of AD versus non-AD findings based on reviewing the unmodified grey scale images. The choice was recorded. Readers were then asked to rate their confidence in this diagnosis on a scale from 1 to 10. Afterwards, the size rank colour overlay was made available to be turned on and off as required by the reader. Diagnostic confidence was recorded again to determine the impact of the added information. The overlay method was classified as *useful *for cases where it confirmed an initially correct assessment, and for cases where it reduced confidence in an initially incorrect assessment. If confidence in a correct assessment was reduced, or if confidence in an incorrect assessment was increased, the overlay method was rated as *not useful *for the case in question. An *unchanged *verdict was recorded if readers recorded no change in confidence.

## Results

Individual results of all 28 classifications made are shown in Table 1. Of these, 22 were correct (80 %). The size rank colour overlays were *useful *in 18 of 28 cases, as determined by their impact on readers' diagnostic confidence. A *not useful *result was found in 6 of 28 cases. The difference in confidence in *useful *results was 1 in 11/18 cases, 2 in 5/18 cases, and 4 in 2/18 cases. The difference in confidence in *not useful *results was 1 in 5/6 cases and 2 in 1/6 cases. The impact of the overlay on diagnostic confidence was significant as measured by a two-tailed Wilcoxon signed-rank test (*p *< 0.02).

**Table 1:** Per-case results

	Subject	Confidence before	After	Change	Diagnosis correct	Effect helpful	Neutral	Unhelpful
Reader 1	3	8	4	4	False	1	0	0
	7	7	5	-2	true	0	0	1
	12	8	8	0	true	0	1	0
	14	8	4	4	false	1	0	0
	17	7	8	1	true	1	0	0
	19	7	8	1	true	1	0	0
	20	6	8	2	true	1	0	0
	27	7	6	1	false	1	0	0
	29	8	8	0	true	0	1	0
	31	7	7	0	true	0	1	0
	32	6	7	-1	false	0	0	1
	33	7	7	0	false	0	1	0
	35	6	8	2	true	1	0	0
	37	7	8	1	true	1	0	0

Reader 2	3	9	10	1	true	1	0	0
	7	9	8	-1	true	0	0	1
	12	8	9	1	true	1	0	0
	14	8	9	1	true	1	0	0
	17	8	9	1	true	1	0	0
	19	8	9	1	true	1	0	0
	20	8	9	1	true	1	0	0
	27	7	9	2	true	1	0	0
	29	8	7	-1	true	0	0	1
	31	7	9	2	true	1	0	0
	32	8	9	-1	false	0	0	1
	33	6	8	2	true	1	0	0
	35	9	10	1	true	1	0	0
	37	8	7	-1	true	0	0	1

Sum				22		18	4	6

Results by diagnosis and type of influence on diagnostic confidence (14 subjects rated by two readers) are shown in Table 2.

**Table 2:** Influence of overlay information on diagnostic confidence.

	Confidence		
	Increased	Unchanged	Reduced
Diagnosis correct	15	3	4
Diagnosis wrong	2	1	3

In cases of AD where the initial assessment was correct, the readers' confidence was increased particularly if the overlay indicated a relatively small cortex, a small hippocampus or large lateral ventricle on at least one side. Readers noted that in AD the overlay looked strongly asymmetrical, especially in the cortices, and even when the grey scale findings did not suggest a large amount of anatomical asymmetry. Asymmetry of the overlay was also seen in some of the normal subjects.

In two of the subjects with AD, the cortex size was shown to rank high. Both of these subjects had wide sulcal spaces that were mislabelled as part of the cortex by the label propagation process. In another case of AD, massively large lateral ventricles were mislabelled as white matter. One observer rated these misregistered overlays as reducing confidence, the other observer disregarded the information pertaining to the misregistered structures and considered only the remaining, correctly registered structures.

## Discussion

This study demonstrates the utility of providing automatically derived comparative volumetric information in distinguishing Alzheimer disease from normal ageing on MRI images of the brain. Presenting this information as a colour overlay on the grey scale sectional images improved the diagnostic outcome. The fact that volumetry was performed in an automated fashion using pre-prepared expert input indicates that the method is scalable to larger cohorts.

The results are novel in that they provide proof of concept for diagnostic decision support based on brain volumetry. In the majority of cases, we found that the colour overlays confirmed readers' initial assessment of the plain grey scale MR image. In a smaller number of cases, readers made an erroneous classification and stated that, after reviewing the colour-overlaid version of the image, they were less confident in this assessment. In a practical setting, they might have sought further information or advice from a more senior diagnostician or a specialist, which would have been a desirable outcome.

Problematic situations arise when misregistration results in erroneous estimation of the volumes of ROIs. Some refinements of the automatic segmentation method would help reduce this problem. For example, fusing multiple manual atlases [11] and atlas selection [12] can increase segmentation accuracy substantially. Choosing more accurate or specialized atlases as label sources is another avenue towards increasing the potential benefit of the approaach. In the present work, the input segmentation was based on 43 regions. By using atlases based on a stricter protocol using more and smaller ROIs [13], more as well as more accurate information could be used to generate the colour overlay. In [14], we showed that such atlases and a label propagation-fusion approach provide sufficient volumetric information to distinguish normal from atrophic hippocampi without user intervention.

Training readers in the use of volumetry overlays is likely to improve outcomes, as they will anticipate and recognize situations where labels might be inaccurate, for example when subject pathology prevents correct segmentation. Nevertheless, the surfeit of *useful *over *not useful *outcomes in this study indicates that even coarse comparative volumetry can provide a benefit for diagnosis.

For this proof-of-concept work, we chose a subjective measure – diagnostic confidence – and assessed the difference before and after presentation of the overlay. The results encourage a study on larger numbers of subjects and readers, which would allow an approach where accuracy of the forced choice is used as the outcome measure. Using larger cohorts, it will be possible to eliminate the subjectivity of the approach presented here, as well as potential bias resulting from readers' expectations or desire for a particular outcome of the study by presenting additional subgroups with "mock" overlays containing randomly assigned colours.

The small number of subjects used for this study also meant that no stratification according to the severity of disease-related change in the subjects was possible. Comparative volumetry would be particularly useful if its impact was present in patients with early disease, where interrater variability is particularly high [4]. We aim to address this question in future work.

Another limitation of this study is that readers were merely offered an explanation of how the overlay was generated, while no examples were shown and no opportunity for practice was given. As readers learn to integrate the extra information provided by the overlay, the influence on their diagnostic decision is likely to evolve. Again, given larger subject numbers, it would have been possible to include a practice phase in the study design, which would have minimized any distortion of the results arising from readers' skill development in using the colour overlays.

## Conclusion

The results of this study indicate that an integrated presentation of comparative volumetry results can support diagnostic decision making in imaging of neurodegenerative disease.

Outside the realm of clinical imaging, similar tools could aid drug discovery by providing better surrogate endpoints for measuring effects of candidate treatments [15]. In imaging research, the possibility of uncovering structural change or patterns of change too subtle to detect visually could aid in the detection of patterns of progression, risk factors and prognostic indicators [16].

## Competing interests

The authors declare that they have no competing interests.

## Authors' contributions

RAH: Conception, design, data acquisition, analysis, interpretation, manuscript draft and revision. JVH: Conception, analysis, interpretation, manuscript revision. DR: Conception, design, manuscript revision. CJH:  Data acquisition, analysis, manuscript revision. RIA: Data acquisition, analysis, manuscript revision. AH: Conception, analysis, interpretation, manuscript revision.

## Pre-publication history

The pre-publication history for this paper can be accessed here:


